# Bis{μ-2-[(4,6-bis{(2-hydroxy-5-methylphenyl)[(pyridin-2-yl)methyl]amino}-1,3,5-triazin-2-yl)[(pyridin-2-yl-κ*N*)methyl]amino-κ*N*]-4-methyl­phenolato-1:2κ^2^ *O*:*O*}bis­[(nitrato-κ^2^ *O*,*O*′)zinc]–acetonitrile–water (2/4/1)

**DOI:** 10.1107/S1600536811053451

**Published:** 2012-01-21

**Authors:** Palanisami Uma Maheswari, Simon J. Teat, Olivier Roubeau, Jan Reedijk

**Affiliations:** a Leiden Institute of Chemistry, Leiden University, PO Box 9502, 2300 RA, Leiden, The Netherlands; b Advanced Light Source, Lawrence Berkeley, National Laboratory, Berkeley, California 94720, USA; c Instituto de Ciencia de Materiales de Aragon, CSIC and Universidad de Zaragoza, Plaza San Francisco s/n, 50009 Zaragoza, Spain; d Department of Chemistry, King Saud University, PO Box 2455, Riyadh, 11451, Saudi Arabia

## Abstract

The title compound, [Zn_2_(C_42_H_38_N_9_O_3_)_2_(NO_3_)_2_]·2CH_3_CN·0.5H_2_O, is a bis-phenolate-bridged dinuclear Zn^II^ complex. The asymmetric unit comprises half the zinc complex (the full complex is completed by the application of a centre of inversion), one acetonitrile solvent mol­ecule and a quarter of a water mol­ecule (located on a twofold axis with half-occupancy; H atoms were not located for this mol­ecule). Each triazine-based multidentate ligand uses a phenolate group to bridge Zn^II^ ions, generating a Zn_2_O_2_ core. The Zn^II^ ions are five-coordinate, with an additional long Zn—O contact [2.6465 (16) Å], and include a semi-bidentate nitrate ion and a *N*,*N*′,*O*-tridentate mode of the ligand in the coordination sphere. Non-coordinating pyridine groups form intra­molecular O—H⋯N hydrogen bonds with phenol groups. As suggested by the short O⋯O donor–acceptor distances between the disordered water molecules and phenol O atoms, these groups also participate in hydrogen bonding.

## Related literature

For a related structure, see: Maheswari *et al.* (2007 ▶). For the synthesis of the ligand, see: de Hoog *et al.* (2002 ▶); Gamez *et al.* (2003 ▶). For a description of the geometry of complexes with five-coordinate metal atoms, see: Addison *et al.* (1984 ▶).
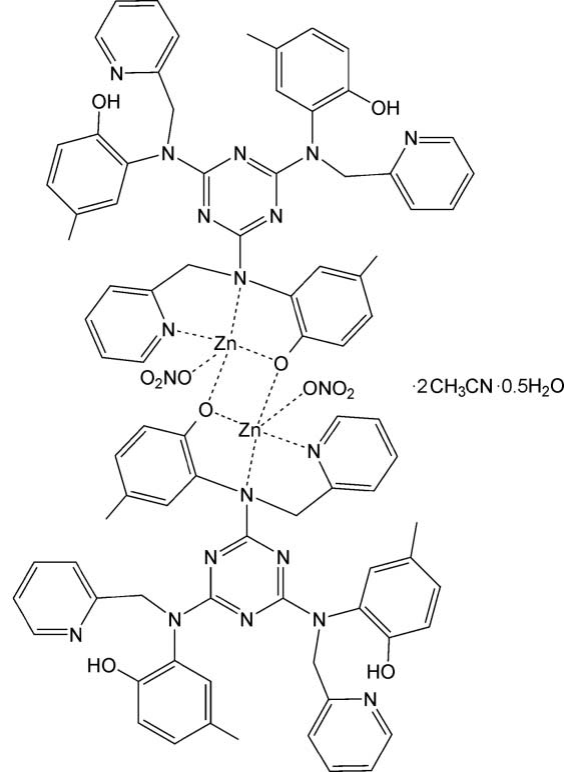


## Experimental

### 

#### Crystal data

[Zn_2_(C_42_H_38_N_9_O_3_)_2_(NO_3_)_2_]·2C_2_H_3_N·0.5H_2_O
*M* *_r_* = 1779.53
Monoclinic, 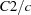

*a* = 31.154 (3) Å
*b* = 15.3768 (13) Å
*c* = 18.0060 (16) Å
β = 100.253 (2)°
*V* = 8488.0 (13) Å^3^
*Z* = 4
Synchrotron radiation
λ = 0.68940 Å
μ = 0.59 mm^−1^
*T* = 150 K
0.16 × 0.04 × 0.04 mm

#### Data collection

Bruker APEXII CCD diffractometer
Absorption correction: multi-scan (*SADABS*; Sheldrick, 1996 ▶) *T*_min_ = 0.87, *T*_max_ = 0.93
47083 measured reflections
12791 independent reflections
10387 reflections with *I* > 2σ(*I*)
*R*_int_ = 0.039

#### Refinement

*R*[*F* ^2^ > 2σ(*F* ^2^)] = 0.039
*wR*(*F* ^2^) = 0.108
*S* = 1.06
12791 reflections
570 parameters
H-atom parameters constrained
Δρ_max_ = 0.56 e Å^−3^
Δρ_min_ = −0.37 e Å^−3^

### 

Data collection: *APEX2* (Bruker, 2009 ▶); cell refinement: *SAINT* (Bruker, 2007 ▶); data reduction: *SAINT*; program(s) used to solve structure: *SHELXTL* (Sheldrick, 2008 ▶); program(s) used to refine structure: *SHELXTL*; molecular graphics: *SHELXTL*; software used to prepare material for publication: *SHELXTL*.

## Supplementary Material

Crystal structure: contains datablock(s) I, global. DOI: 10.1107/S1600536811053451/tk5033sup1.cif

Structure factors: contains datablock(s) I. DOI: 10.1107/S1600536811053451/tk5033Isup2.hkl

Additional supplementary materials:  crystallographic information; 3D view; checkCIF report

## Figures and Tables

**Table 1:** Selected bond lengths (Å)

Zn1—O1	1.9752 (10)
Zn1—O1^i^	2.0386 (10)
Zn1—O10	1.9743 (12)
Zn1—N4	2.4705 (12)
Zn1—N5	2.0066 (13)

Symmetry code: (i) 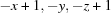.

**Table 2:** Hydrogen-bond geometry (Å, °)

*D*—H⋯*A*	*D*—H	H⋯*A*	*D*⋯*A*	*D*—H⋯*A*
O2—H2⋯N7	0.84	1.93	2.745 (2)	164
O3—H3⋯N9	0.84	1.87	2.688 (2)	164
O1w⋯O3			2.676 (3)	
O1w⋯O3^ii^			2.676 (3)	

Symmetry code: (ii) 
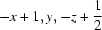
.

**Fig. 1.**
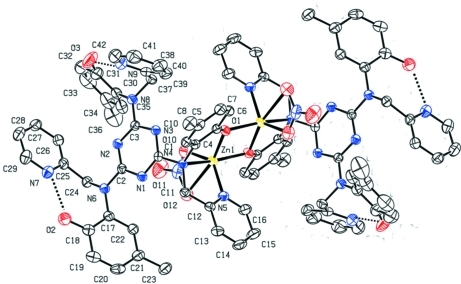
Molecular structure of [Zn2(H2L)2(NO3)2](CH3CN)2(H2O)0.5 showing the intramolecular O—H···N hydrogen bonding as dashed lines. Hydrogen atoms and lattice molecules have been omitted for clarity. Unlabelled atoms are related by symmetry operation 1-x, -y, 1-z.

## supplementary crystallographic information

### Comment

The ligand 4,4',4''-trimethyl-2,2',2''-[(1,3,5-triazine-2,4,6-triyl)tris{[(pyridin-2-yl)methyl]imino}]triphenol is a potential multidentate ligand towards metal ions. Only with zinc nitrate was a reproducible crystalline compound obtained, and its three-dimensional structure is reported here. The resulting geometry for the zinc(II) ion, Fig. 1 and Table 1, is not unusual and resembles that of an earlier reported smaller fragment 4-methyl-2-[(2-pyridylmethyl)amino]phenol(Maheswari *et al.*, 2007). The acetonitrile molecules fill the lattice space, as does a water molecule with 50% occupancy, albeit it H-bonded to 2 phenols, Table 2. The coordination around zinc is distorted square pyramidal, with a τ value (Addison *et al.*, 1984) of 0.33; in addition a semi-coordinating oxygen from a chelating nitrate is present at 2.6465 (16) Å [Zn1···O12].

### Experimental

The ligand 4,4',4''-trimethyl-2,2',2''-[(1,3,5-triazine-2,4,6-triyl)tris{[(pyridin-2-yl)methyl]imino}]triphenol was prepared by reacting 2,4,6-trichloro-1,3,5-triazine with 4-methyl-2-{[(pyridine-2-yl)methyl]amino}phenol in MeOH in the ratio 1:3, as described earlier for similar compounds (de Hoog *et al.*, 2002; Gamez *et al.*, 2003). The ligand (1 mmol, 0.720 g) was dissolved in warm acetonitrile and Zn(NO_3_)_2_ (H_2_O)_3_ (1 mmol, 0.2434 g), also dissolved in warm acetonitrile, was added drop wise to the solution of the ligand under stirring and then filtered. The resulting clear solution is colourless and light-yellow crystals were harvested after a week.

### Refinement

Carbon-bound H-atoms were placed in calculated positions (C—H 0.95 to 0.99 Å) and were included in the refinement in the riding model approximation, with *U*_iso_(H) set to 1.2 to 1.5*U*_equiv_(C). Hydrogen atoms could not be found or placed on O1w and were therefore not included the refinement. Significant Hirshfeld differences were noted for the N10—O10 and C33—C34 bonds. As the atom types are correct, these are probably due to the a rocking "motion" of the groups in question.

### Crystal data

**Table:**

[Zn_2_(C_42_H_38_N_9_O_3_)_2_(NO_3_)_2_]·2C_2_H_3_N·0.5H_2_O	*F*(000) = 3696
*M**_r_* = 1779.53	*D*_x_ = 1.393 Mg m^−^^3^
Monoclinic, *C*2/*c*	Synchrotron radiation, λ = 0.68940 Å
Hall symbol: -C 2yc	Cell parameters from 7351 reflections
*a* = 31.154 (3) Å	θ = 2.8–29.4°
*b* = 15.3768 (13) Å	µ = 0.59 mm^−^^1^
*c* = 18.0060 (16) Å	*T* = 150 K
β = 100.253 (2)°	Parallelpiped, light-yellow
*V* = 8488.0 (13) Å^3^	0.16 × 0.04 × 0.04 mm
*Z* = 4	

### Data collection

**Table:**

Bruker APEXII CCD diffractometer	12791 independent reflections
Radiation source: Daresbury SRS station 9.8	10387 reflections with *I* > 2σ(*I*)
silicon 111	*R*_int_ = 0.039
ω rotation with narrow frames scans	θ_max_ = 29.5°, θ_min_ = 2.6°
Absorption correction: multi-scan (*SADABS*; Sheldrick, 1996)	*h* = −43→44
*T*_min_ = 0.87, *T*_max_ = 0.93	*k* = −21→21
47083 measured reflections	*l* = −25→25

### Refinement

**Table:**

Refinement on *F*^2^	Primary atom site location: structure-invariant direct methods
Least-squares matrix: full	Secondary atom site location: difference Fourier map
*R*[*F*^2^ > 2σ(*F*^2^)] = 0.039	Hydrogen site location: inferred from neighbouring sites
*wR*(*F*^2^) = 0.108	H-atom parameters constrained
*S* = 1.06	*w* = 1/[σ^2^(*F*_o_^2^) + (0.0554*P*)^2^ + 3.4344*P*] where *P* = (*F*_o_^2^ + 2*F*_c_^2^)/3
12791 reflections	(Δ/σ)_max_ = 0.001
570 parameters	Δρ_max_ = 0.56 e Å^−^^3^
0 restraints	Δρ_min_ = −0.37 e Å^−^^3^

### Special details

**Table:**

Geometry. Also a short hydrogen bond is seen for the half-occupied water to two phenol groups: O3—Ow 2.676 (3) Å.All e.s.d.'s (except the e.s.d. in the dihedral angle between two l.s. planes) are estimated using the full covariance matrix. The cell e.s.d.'s are taken into account individually in the estimation of e.s.d.'s in distances, angles and torsion angles; correlations between e.s.d.'s in cell parameters are only used when they are defined by crystal symmetry. An approximate (isotropic) treatment of cell e.s.d.'s is used for estimating e.s.d.'s involving l.s. planes.
Refinement. Refinement of *F*^2^ against ALL reflections. The weighted *R*-factor *wR* and goodness of fit *S* are based on *F*^2^, conventional *R*-factors *R* are based on *F*, with *F* set to zero for negative *F*^2^. The threshold expression of *F*^2^ > σ(*F*^2^) is used only for calculating *R*-factors(gt) *etc*. and is not relevant to the choice of reflections for refinement. *R*-factors based on *F*^2^ are statistically about twice as large as those based on *F*, and *R*- factors based on ALL data will be even larger.

### Fractional atomic coordinates and isotropic or equivalent isotropic displacement parameters (Å^2^)

**Table:**

	*x*	*y*	*z*	*U*_iso_*/*U*_eq_	Occ. (<1)
Zn1	0.458384 (5)	0.041228 (10)	0.518223 (9)	0.02346 (5)	
C1	0.44079 (4)	0.23008 (8)	0.54547 (7)	0.0209 (2)	
N1	0.40078 (4)	0.24336 (8)	0.55736 (7)	0.0243 (2)	
C2	0.37475 (4)	0.28245 (9)	0.49918 (8)	0.0229 (2)	
N2	0.38661 (4)	0.30793 (8)	0.43508 (7)	0.0246 (2)	
C3	0.42888 (4)	0.29428 (9)	0.43300 (8)	0.0231 (2)	
N3	0.45848 (4)	0.25762 (8)	0.48758 (6)	0.0231 (2)	
N4	0.46675 (4)	0.17411 (7)	0.59659 (6)	0.0215 (2)	
C4	0.51379 (4)	0.18675 (9)	0.60775 (7)	0.0222 (2)	
C5	0.53898 (4)	0.13243 (9)	0.57023 (8)	0.0239 (3)	
O1	0.52016 (3)	0.07228 (7)	0.52085 (6)	0.0254 (2)	
C6	0.58419 (5)	0.14437 (10)	0.58511 (9)	0.0293 (3)	
H6A	0.6021	0.1098	0.5594	0.035*	
C7	0.60321 (5)	0.20596 (10)	0.63692 (9)	0.0331 (3)	
H7A	0.6341	0.2114	0.6472	0.040*	
C8	0.57822 (5)	0.25999 (10)	0.67413 (9)	0.0307 (3)	
C9	0.53309 (5)	0.25013 (9)	0.65745 (8)	0.0261 (3)	
H9A	0.5152	0.2876	0.6806	0.031*	
C10	0.59920 (6)	0.32631 (12)	0.73076 (11)	0.0418 (4)	
H10A	0.6266	0.3029	0.7589	0.063*	
H10B	0.5794	0.3398	0.7659	0.063*	
H10C	0.6052	0.3794	0.7043	0.063*	
C11	0.44937 (5)	0.14906 (9)	0.66421 (8)	0.0244 (3)	
H11A	0.4195	0.1728	0.6600	0.029*	
H11B	0.4676	0.1762	0.7088	0.029*	
C12	0.44780 (4)	0.05271 (9)	0.67719 (8)	0.0251 (3)	
C13	0.44166 (5)	0.02155 (11)	0.74728 (9)	0.0336 (3)	
H13A	0.4400	0.0607	0.7875	0.040*	
C14	0.43808 (7)	−0.06705 (13)	0.75745 (11)	0.0463 (4)	
H14A	0.4340	−0.0894	0.8049	0.056*	
C15	0.44043 (8)	−0.12283 (12)	0.69819 (12)	0.0512 (5)	
H15A	0.4379	−0.1839	0.7040	0.061*	
C16	0.44652 (6)	−0.08789 (11)	0.63057 (11)	0.0418 (4)	
H16A	0.4483	−0.1260	0.5897	0.050*	
N5	0.45009 (4)	−0.00152 (8)	0.62001 (7)	0.0282 (2)	
N6	0.33232 (4)	0.29589 (8)	0.50585 (7)	0.0258 (2)	
O2	0.29522 (4)	0.41085 (8)	0.60279 (7)	0.0409 (3)	
H2	0.3006	0.4215	0.5596	0.061*	
C17	0.31620 (4)	0.26517 (10)	0.57091 (8)	0.0274 (3)	
C18	0.29838 (5)	0.32432 (11)	0.61599 (9)	0.0311 (3)	
C19	0.28357 (5)	0.29258 (12)	0.67969 (9)	0.0374 (4)	
H19A	0.2702	0.3313	0.7099	0.045*	
C20	0.28802 (5)	0.20607 (13)	0.69934 (9)	0.0391 (4)	
H20A	0.2785	0.1867	0.7438	0.047*	
C21	0.30606 (5)	0.14642 (12)	0.65563 (10)	0.0377 (4)	
C22	0.31922 (5)	0.17715 (11)	0.59040 (9)	0.0322 (3)	
H22A	0.3305	0.1373	0.5584	0.039*	
C23	0.31110 (7)	0.05114 (14)	0.67629 (14)	0.0536 (5)	
H23A	0.3254	0.0455	0.7291	0.080*	
H23B	0.3289	0.0225	0.6437	0.080*	
H23C	0.2823	0.0237	0.6694	0.080*	
C24	0.30153 (4)	0.31816 (10)	0.43693 (8)	0.0281 (3)	
H24A	0.2715	0.3083	0.4459	0.034*	
H24B	0.3064	0.2785	0.3960	0.034*	
C25	0.30527 (4)	0.41092 (10)	0.41109 (9)	0.0287 (3)	
C26	0.30984 (5)	0.43047 (11)	0.33770 (9)	0.0332 (3)	
H26A	0.3124	0.3852	0.3028	0.040*	
C27	0.31063 (6)	0.51666 (12)	0.31593 (11)	0.0389 (4)	
H27A	0.3136	0.5315	0.2659	0.047*	
C28	0.30703 (6)	0.58063 (12)	0.36824 (11)	0.0418 (4)	
H28A	0.3070	0.6403	0.3547	0.050*	
C29	0.30344 (6)	0.55613 (11)	0.44057 (11)	0.0419 (4)	
H29A	0.3016	0.6004	0.4767	0.050*	
N7	0.30247 (5)	0.47250 (9)	0.46250 (8)	0.0351 (3)	
N8	0.44353 (4)	0.31853 (8)	0.36930 (7)	0.0279 (2)	
C30	0.41349 (5)	0.33489 (11)	0.30045 (8)	0.0339 (3)	
C31	0.41664 (7)	0.41154 (14)	0.26101 (11)	0.0478 (5)	
O3	0.44543 (6)	0.47581 (11)	0.28450 (11)	0.0692 (5)	
H3	0.4637	0.4584	0.3215	0.104*	
C32	0.38783 (8)	0.42351 (19)	0.19253 (13)	0.0664 (7)	
H32A	0.3895	0.4752	0.1643	0.080*	
C33	0.35731 (8)	0.3616 (2)	0.16589 (12)	0.0682 (7)	
H33A	0.3383	0.3715	0.1193	0.082*	
C34	0.35332 (7)	0.28482 (16)	0.20493 (11)	0.0535 (5)	
C35	0.38230 (6)	0.27290 (13)	0.27274 (10)	0.0404 (4)	
H35A	0.3807	0.2210	0.3007	0.048*	
C36	0.31979 (9)	0.2168 (2)	0.17639 (15)	0.0825 (9)	
H36A	0.3246	0.1954	0.1272	0.124*	
H36B	0.2906	0.2422	0.1710	0.124*	
H36C	0.3224	0.1683	0.2123	0.124*	
C37	0.48971 (5)	0.30312 (10)	0.36586 (9)	0.0306 (3)	
H37A	0.4940	0.3087	0.3129	0.037*	
H37B	0.4975	0.2429	0.3826	0.037*	
C38	0.51987 (5)	0.36585 (10)	0.41449 (9)	0.0313 (3)	
C39	0.55425 (5)	0.33806 (12)	0.46840 (10)	0.0383 (4)	
H39A	0.5595	0.2778	0.4771	0.046*	
C40	0.58092 (6)	0.39966 (13)	0.50970 (12)	0.0478 (4)	
H40A	0.6047	0.3822	0.5473	0.057*	
C41	0.57254 (7)	0.48598 (14)	0.49561 (14)	0.0532 (5)	
H41A	0.5906	0.5294	0.5227	0.064*	
C42	0.53723 (7)	0.50866 (13)	0.44110 (14)	0.0532 (5)	
H42A	0.5312	0.5686	0.4317	0.064*	
N9	0.51116 (5)	0.44982 (9)	0.40105 (9)	0.0405 (3)	
N10	0.38032 (5)	0.07108 (9)	0.43434 (9)	0.0395 (3)	
O10	0.41860 (4)	0.08881 (8)	0.42993 (7)	0.0374 (3)	
O11	0.35018 (5)	0.09345 (12)	0.38307 (11)	0.0715 (5)	
O12	0.37223 (5)	0.03042 (9)	0.49054 (9)	0.0512 (3)	
N1S	0.30136 (12)	0.78257 (19)	0.4472 (2)	0.1195 (12)	
C1S	0.28966 (9)	0.85082 (18)	0.43433 (18)	0.0727 (8)	
C2S	0.27491 (11)	0.9365 (2)	0.4163 (3)	0.1041 (12)	
H2S1	0.2950	0.9657	0.3883	0.156*	
H2S2	0.2737	0.9687	0.4629	0.156*	
H2S3	0.2457	0.9345	0.3851	0.156*	
O1W	0.5000	0.5976 (3)	0.2500	0.0740 (15)	0.50

### Atomic displacement parameters (Å^2^)

**Table:**

	*U*^11^	*U*^22^	*U*^33^	*U*^12^	*U*^13^	*U*^23^
Zn1	0.02159 (8)	0.02630 (9)	0.02305 (9)	0.00057 (6)	0.00550 (6)	−0.00004 (6)
C1	0.0212 (6)	0.0200 (5)	0.0206 (6)	0.0003 (4)	0.0016 (4)	−0.0014 (4)
N1	0.0206 (5)	0.0284 (6)	0.0237 (6)	0.0014 (4)	0.0032 (4)	0.0023 (4)
C2	0.0206 (6)	0.0217 (6)	0.0260 (6)	0.0006 (5)	0.0027 (5)	−0.0007 (5)
N2	0.0234 (5)	0.0263 (6)	0.0237 (6)	0.0022 (4)	0.0029 (4)	0.0017 (4)
C3	0.0242 (6)	0.0226 (6)	0.0224 (6)	0.0005 (5)	0.0040 (5)	−0.0002 (5)
N3	0.0220 (5)	0.0248 (5)	0.0226 (5)	0.0012 (4)	0.0041 (4)	0.0013 (4)
N4	0.0187 (5)	0.0258 (5)	0.0199 (5)	0.0009 (4)	0.0033 (4)	0.0022 (4)
C4	0.0199 (6)	0.0248 (6)	0.0209 (6)	−0.0010 (5)	0.0011 (4)	0.0020 (5)
C5	0.0221 (6)	0.0242 (6)	0.0248 (6)	−0.0013 (5)	0.0028 (5)	0.0031 (5)
O1	0.0223 (4)	0.0265 (5)	0.0281 (5)	−0.0018 (4)	0.0062 (4)	−0.0038 (4)
C6	0.0221 (6)	0.0295 (7)	0.0365 (8)	0.0004 (5)	0.0057 (5)	0.0034 (6)
C7	0.0215 (6)	0.0353 (8)	0.0403 (8)	−0.0053 (6)	−0.0007 (6)	0.0060 (6)
C8	0.0301 (7)	0.0297 (7)	0.0295 (7)	−0.0071 (6)	−0.0024 (6)	0.0034 (6)
C9	0.0275 (7)	0.0249 (6)	0.0244 (7)	−0.0010 (5)	0.0010 (5)	0.0015 (5)
C10	0.0387 (9)	0.0403 (9)	0.0417 (9)	−0.0125 (7)	−0.0060 (7)	−0.0029 (7)
C11	0.0255 (6)	0.0287 (7)	0.0192 (6)	0.0006 (5)	0.0048 (5)	0.0007 (5)
C12	0.0216 (6)	0.0301 (7)	0.0241 (6)	−0.0010 (5)	0.0050 (5)	0.0005 (5)
C13	0.0370 (8)	0.0387 (8)	0.0274 (7)	−0.0039 (6)	0.0115 (6)	0.0012 (6)
C14	0.0649 (12)	0.0420 (9)	0.0384 (9)	−0.0031 (9)	0.0265 (9)	0.0099 (8)
C15	0.0794 (14)	0.0304 (8)	0.0531 (12)	−0.0030 (9)	0.0369 (11)	0.0074 (8)
C16	0.0603 (11)	0.0284 (8)	0.0436 (10)	−0.0030 (7)	0.0278 (8)	−0.0004 (7)
N5	0.0314 (6)	0.0267 (6)	0.0286 (6)	−0.0011 (5)	0.0113 (5)	0.0007 (5)
N6	0.0188 (5)	0.0311 (6)	0.0271 (6)	0.0016 (4)	0.0028 (4)	0.0047 (5)
O2	0.0510 (7)	0.0345 (6)	0.0394 (7)	−0.0020 (5)	0.0145 (6)	−0.0057 (5)
C17	0.0184 (6)	0.0332 (7)	0.0298 (7)	−0.0013 (5)	0.0020 (5)	0.0036 (6)
C18	0.0264 (7)	0.0370 (8)	0.0287 (7)	−0.0048 (6)	0.0016 (5)	−0.0025 (6)
C19	0.0325 (8)	0.0511 (10)	0.0287 (8)	−0.0092 (7)	0.0060 (6)	−0.0064 (7)
C20	0.0289 (8)	0.0590 (11)	0.0284 (8)	−0.0105 (7)	0.0023 (6)	0.0072 (7)
C21	0.0231 (7)	0.0458 (9)	0.0426 (9)	−0.0026 (6)	0.0017 (6)	0.0149 (7)
C22	0.0215 (6)	0.0357 (8)	0.0392 (8)	0.0019 (6)	0.0051 (6)	0.0080 (6)
C23	0.0389 (10)	0.0548 (12)	0.0685 (14)	0.0053 (8)	0.0135 (9)	0.0319 (10)
C24	0.0201 (6)	0.0310 (7)	0.0312 (7)	0.0004 (5)	−0.0007 (5)	0.0043 (6)
C25	0.0202 (6)	0.0304 (7)	0.0334 (8)	0.0026 (5)	−0.0006 (5)	0.0037 (6)
C26	0.0284 (7)	0.0350 (8)	0.0361 (8)	0.0038 (6)	0.0055 (6)	0.0051 (6)
C27	0.0350 (8)	0.0411 (9)	0.0406 (9)	0.0018 (7)	0.0070 (7)	0.0119 (7)
C28	0.0421 (9)	0.0321 (8)	0.0485 (10)	−0.0019 (7)	0.0006 (8)	0.0092 (7)
C29	0.0464 (10)	0.0316 (8)	0.0451 (10)	−0.0003 (7)	0.0014 (8)	0.0000 (7)
N7	0.0368 (7)	0.0328 (7)	0.0340 (7)	0.0005 (5)	0.0021 (6)	0.0036 (5)
N8	0.0279 (6)	0.0340 (6)	0.0224 (6)	0.0060 (5)	0.0065 (4)	0.0046 (5)
C30	0.0343 (8)	0.0455 (9)	0.0224 (7)	0.0131 (7)	0.0067 (6)	0.0043 (6)
C31	0.0452 (10)	0.0592 (12)	0.0395 (10)	0.0119 (9)	0.0089 (8)	0.0206 (9)
O3	0.0639 (10)	0.0611 (10)	0.0773 (12)	0.0008 (8)	−0.0022 (9)	0.0417 (9)
C32	0.0593 (14)	0.0960 (19)	0.0440 (12)	0.0224 (13)	0.0092 (10)	0.0364 (12)
C33	0.0544 (13)	0.120 (2)	0.0269 (9)	0.0290 (14)	−0.0009 (8)	0.0068 (11)
C34	0.0477 (11)	0.0789 (15)	0.0306 (9)	0.0197 (10)	−0.0023 (8)	−0.0115 (9)
C35	0.0397 (9)	0.0509 (10)	0.0287 (8)	0.0133 (7)	0.0014 (6)	−0.0050 (7)
C36	0.0671 (16)	0.108 (2)	0.0594 (15)	0.0119 (15)	−0.0233 (12)	−0.0309 (15)
C37	0.0304 (7)	0.0370 (8)	0.0266 (7)	0.0062 (6)	0.0109 (6)	0.0033 (6)
C38	0.0315 (7)	0.0340 (7)	0.0318 (8)	−0.0005 (6)	0.0145 (6)	0.0041 (6)
C39	0.0335 (8)	0.0372 (8)	0.0441 (9)	−0.0057 (6)	0.0065 (7)	0.0077 (7)
C40	0.0394 (9)	0.0517 (11)	0.0507 (11)	−0.0141 (8)	0.0034 (8)	0.0045 (9)
C41	0.0501 (11)	0.0463 (10)	0.0660 (14)	−0.0170 (9)	0.0178 (10)	−0.0099 (10)
C42	0.0546 (12)	0.0328 (9)	0.0757 (15)	−0.0051 (8)	0.0209 (11)	−0.0002 (9)
N9	0.0421 (8)	0.0330 (7)	0.0493 (9)	0.0022 (6)	0.0158 (7)	0.0056 (6)
N10	0.0492 (9)	0.0289 (7)	0.0376 (8)	0.0086 (6)	−0.0001 (6)	0.0016 (6)
O10	0.0399 (6)	0.0382 (6)	0.0310 (6)	0.0015 (5)	−0.0020 (5)	−0.0014 (5)
O11	0.0415 (8)	0.0744 (11)	0.0861 (12)	−0.0037 (7)	−0.0226 (8)	0.0281 (9)
O12	0.0466 (8)	0.0519 (8)	0.0579 (9)	0.0067 (6)	0.0169 (7)	0.0065 (7)
N1S	0.137 (3)	0.0659 (17)	0.172 (3)	0.0061 (17)	0.072 (2)	−0.0190 (19)
C1S	0.0600 (14)	0.0625 (16)	0.105 (2)	−0.0031 (12)	0.0395 (15)	−0.0205 (15)
C2S	0.0685 (19)	0.0724 (19)	0.175 (4)	0.0003 (15)	0.031 (2)	−0.005 (2)
O1W	0.096 (4)	0.046 (2)	0.094 (4)	0.000	0.052 (3)	0.000

### Geometric parameters (Å, °)

**Table:**

Zn1—Zn1^i^	3.0607 (4)	C21—C23	1.513 (3)
Zn1—O1	1.9752 (10)	C22—H22A	0.9500
Zn1—O1^i^	2.0386 (10)	C23—H23A	0.9800
Zn1—O10	1.9743 (12)	C23—H23B	0.9800
Zn1—N4	2.4705 (12)	C23—H23C	0.9800
Zn1—N5	2.0066 (13)	C24—H24A	0.9900
C1—N1	1.3176 (17)	C24—H24B	0.9900
C1—N3	1.3320 (17)	C24—C25	1.511 (2)
C1—N4	1.4061 (16)	C25—C26	1.387 (2)
N1—C2	1.3467 (17)	C25—N7	1.338 (2)
C2—N2	1.3319 (18)	C26—H26A	0.9500
C2—N6	1.3642 (17)	C26—C27	1.384 (2)
N2—C3	1.3405 (17)	C27—H27A	0.9500
C3—N3	1.3460 (17)	C27—C28	1.380 (3)
C3—N8	1.3598 (18)	C28—H28A	0.9500
N4—C4	1.4563 (16)	C28—C29	1.379 (3)
N4—C11	1.4693 (17)	C29—H29A	0.9500
C4—C5	1.3994 (19)	C29—N7	1.347 (2)
C4—C9	1.3860 (19)	N8—C30	1.4363 (19)
C5—O1	1.3433 (17)	N8—C37	1.4704 (19)
C5—C6	1.3982 (19)	C30—C31	1.389 (3)
O1—Zn1^i^	2.0386 (10)	C30—C35	1.389 (3)
C6—H6A	0.9500	C31—O3	1.351 (3)
C6—C7	1.386 (2)	C31—C32	1.402 (3)
C7—H7A	0.9500	O3—H3	0.8400
C7—C8	1.390 (2)	C32—H32A	0.9500
C8—C9	1.393 (2)	C32—C33	1.370 (4)
C8—C10	1.507 (2)	C33—H33A	0.9500
C9—H9A	0.9500	C33—C34	1.391 (4)
C10—H10A	0.9800	C34—C35	1.395 (2)
C10—H10B	0.9800	C34—C36	1.503 (4)
C10—H10C	0.9800	C35—H35A	0.9500
C11—H11A	0.9900	C36—H36A	0.9800
C11—H11B	0.9900	C36—H36B	0.9800
C11—C12	1.502 (2)	C36—H36C	0.9800
C12—C13	1.395 (2)	C37—H37A	0.9900
C12—N5	1.3367 (19)	C37—H37B	0.9900
C13—H13A	0.9500	C37—C38	1.512 (2)
C13—C14	1.382 (2)	C38—C39	1.379 (2)
C14—H14A	0.9500	C38—N9	1.333 (2)
C14—C15	1.381 (3)	C39—H39A	0.9500
C15—H15A	0.9500	C39—C40	1.386 (3)
C15—C16	1.375 (2)	C40—H40A	0.9500
C16—H16A	0.9500	C40—C41	1.368 (3)
C16—N5	1.349 (2)	C41—H41A	0.9500
N6—C17	1.4342 (19)	C41—C42	1.382 (3)
N6—C24	1.4677 (18)	C42—H42A	0.9500
O2—H2	0.8400	C42—N9	1.338 (3)
O2—C18	1.352 (2)	N10—O10	1.240 (2)
C17—C18	1.398 (2)	N10—O11	1.2425 (19)
C17—C22	1.397 (2)	N10—O12	1.253 (2)
C18—C19	1.398 (2)	N1S—C1S	1.122 (4)
C19—H19A	0.9500	C1S—C2S	1.414 (4)
C19—C20	1.377 (3)	C2S—H2S1	0.9800
C20—H20A	0.9500	C2S—H2S2	0.9800
C20—C21	1.391 (3)	C2S—H2S3	0.9800
C21—C22	1.394 (2)		
			
Zn1^i^—Zn1—N4	117.24 (3)	C19—C20—C21	121.61 (15)
Zn1^i^—Zn1—O1	41.08 (3)	H20A—C20—C21	119.2
Zn1^i^—Zn1—O1^i^	39.55 (3)	C20—C21—C22	117.54 (16)
Zn1^i^—Zn1—N5	108.17 (4)	C20—C21—C23	122.29 (17)
Zn1^i^—Zn1—O10	114.54 (4)	C22—C21—C23	120.17 (18)
N4—Zn1—O1	77.47 (4)	C17—C22—C21	121.56 (16)
N4—Zn1—O1^i^	153.84 (4)	C17—C22—H22A	119.2
N4—Zn1—N5	76.52 (4)	C21—C22—H22A	119.2
N4—Zn1—O10	98.09 (4)	C21—C23—H23A	109.5
O1—Zn1—O1^i^	80.63 (4)	C21—C23—H23B	109.5
O1—Zn1—N5	109.92 (5)	C21—C23—H23C	109.5
O1^i^—Zn1—N5	97.94 (5)	H23A—C23—H23B	109.5
O1—Zn1—O10	113.52 (5)	H23A—C23—H23C	109.5
O1^i^—Zn1—O10	103.70 (5)	H23B—C23—H23C	109.5
N5—Zn1—O10	133.83 (5)	N6—C24—H24A	108.8
N1—C1—N3	127.81 (12)	N6—C24—H24B	108.8
N1—C1—N4	116.05 (12)	N6—C24—C25	113.95 (12)
N3—C1—N4	115.90 (11)	H24A—C24—H24B	107.7
C1—N1—C2	113.50 (12)	H24A—C24—C25	108.8
N1—C2—N2	125.73 (12)	H24B—C24—C25	108.8
N1—C2—N6	117.24 (12)	C24—C25—C26	121.63 (14)
N2—C2—N6	117.02 (12)	C24—C25—N7	115.90 (14)
C2—N2—C3	113.80 (12)	C26—C25—N7	122.43 (14)
N2—C3—N3	126.44 (12)	C25—C26—H26A	120.4
N2—C3—N8	117.36 (12)	C25—C26—C27	119.18 (16)
N3—C3—N8	116.20 (12)	H26A—C26—C27	120.4
C1—N3—C3	112.29 (11)	C26—C27—H27A	120.6
Zn1—N4—C1	98.19 (8)	C26—C27—C28	118.79 (17)
Zn1—N4—C4	100.98 (8)	H27A—C27—C28	120.6
Zn1—N4—C11	103.86 (8)	C27—C28—H28A	120.7
C1—N4—C4	117.14 (11)	C27—C28—C29	118.68 (16)
C1—N4—C11	116.68 (11)	H28A—C28—C29	120.7
C4—N4—C11	115.52 (10)	C28—C29—H29A	118.4
N4—C4—C5	119.39 (12)	C28—C29—N7	123.18 (17)
N4—C4—C9	119.55 (12)	H29A—C29—N7	118.4
C5—C4—C9	121.01 (12)	C25—N7—C29	117.72 (15)
C4—C5—O1	120.94 (12)	C3—N8—C30	120.74 (12)
C4—C5—C6	117.48 (13)	C3—N8—C37	117.98 (12)
O1—C5—C6	121.56 (13)	C30—N8—C37	118.94 (12)
Zn1—O1—Zn1^i^	99.37 (4)	N8—C30—C31	119.71 (16)
Zn1—O1—C5	119.40 (8)	N8—C30—C35	119.77 (15)
Zn1^i^—O1—C5	133.36 (9)	C31—C30—C35	120.50 (16)
C5—C6—H6A	119.5	C30—C31—O3	124.36 (17)
C5—C6—C7	120.94 (14)	C30—C31—C32	117.9 (2)
H6A—C6—C7	119.5	O3—C31—C32	117.69 (19)
C6—C7—H7A	119.2	C31—O3—H3	109.5
C6—C7—C8	121.58 (13)	C31—C32—H32A	119.6
H7A—C7—C8	119.2	C31—C32—C33	120.8 (2)
C7—C8—C9	117.50 (14)	H32A—C32—C33	119.6
C7—C8—C10	121.21 (14)	C32—C33—H33A	119.0
C9—C8—C10	121.29 (15)	C32—C33—C34	122.10 (19)
C4—C9—C8	121.41 (14)	H33A—C33—C34	119.0
C4—C9—H9A	119.3	C33—C34—C35	116.8 (2)
C8—C9—H9A	119.3	C33—C34—C36	122.5 (2)
C8—C10—H10A	109.5	C35—C34—C36	120.7 (2)
C8—C10—H10B	109.5	C30—C35—C34	121.77 (19)
C8—C10—H10C	109.5	C30—C35—H35A	119.1
H10A—C10—H10B	109.5	C34—C35—H35A	119.1
H10A—C10—H10C	109.5	C34—C36—H36A	109.5
H10B—C10—H10C	109.5	C34—C36—H36B	109.5
N4—C11—H11A	108.6	C34—C36—H36C	109.5
N4—C11—H11B	108.6	H36A—C36—H36B	109.5
N4—C11—C12	114.49 (11)	H36A—C36—H36C	109.5
H11A—C11—H11B	107.6	H36B—C36—H36C	109.5
H11A—C11—C12	108.6	N8—C37—H37A	109.1
H11B—C11—C12	108.6	N8—C37—H37B	109.1
C11—C12—C13	119.56 (13)	N8—C37—C38	112.62 (12)
C11—C12—N5	119.20 (12)	H37A—C37—H37B	107.8
C13—C12—N5	121.16 (14)	H37A—C37—C38	109.1
C12—C13—H13A	120.5	H37B—C37—C38	109.1
C12—C13—C14	119.08 (15)	C37—C38—C39	122.31 (15)
H13A—C13—C14	120.5	C37—C38—N9	115.30 (15)
C13—C14—H14A	120.2	C39—C38—N9	122.38 (16)
C13—C14—C15	119.56 (16)	C38—C39—H39A	120.6
H14A—C14—C15	120.2	C38—C39—C40	118.83 (17)
C14—C15—H15A	120.8	H39A—C39—C40	120.6
C14—C15—C16	118.44 (17)	C39—C40—H40A	120.4
H15A—C15—C16	120.8	C39—C40—C41	119.16 (19)
C15—C16—H16A	118.7	H40A—C40—C41	120.4
C15—C16—N5	122.51 (16)	C40—C41—H41A	120.7
H16A—C16—N5	118.7	C40—C41—C42	118.56 (19)
Zn1—N5—C12	122.17 (10)	H41A—C41—C42	120.7
Zn1—N5—C16	118.59 (11)	C41—C42—H42A	118.6
C12—N5—C16	119.25 (13)	C41—C42—N9	122.84 (19)
C2—N6—C17	120.51 (12)	H42A—C42—N9	118.6
C2—N6—C24	117.30 (12)	C38—N9—C42	118.22 (17)
C17—N6—C24	119.75 (11)	O10—N10—O11	120.00 (16)
H2—O2—C18	109.5	O10—N10—O12	119.72 (15)
N6—C17—C18	119.53 (13)	O11—N10—O12	120.29 (17)
N6—C17—C22	120.43 (14)	Zn1—O10—N10	109.88 (10)
C18—C17—C22	120.03 (14)	N1S—C1S—C2S	178.7 (4)
O2—C18—C17	124.20 (14)	C1S—C2S—H2S1	109.5
O2—C18—C19	117.62 (15)	C1S—C2S—H2S2	109.5
C17—C18—C19	118.17 (15)	C1S—C2S—H2S3	109.5
C18—C19—H19A	119.5	H2S1—C2S—H2S2	109.5
C18—C19—C20	121.01 (16)	H2S1—C2S—H2S3	109.5
H19A—C19—C20	119.5	H2S2—C2S—H2S3	109.5
C19—C20—H20A	119.2		
			
N3—C1—N1—C2	6.8 (2)	Zn1^i^—Zn1—N5—C16	−58.76 (13)
N4—C1—N1—C2	−167.31 (11)	N4—Zn1—N5—C12	6.92 (11)
C1—N1—C2—N2	−1.0 (2)	N4—Zn1—N5—C16	−173.40 (13)
C1—N1—C2—N6	177.80 (12)	O1—Zn1—N5—C12	78.01 (12)
N1—C2—N2—C3	−2.4 (2)	O1^i^—Zn1—N5—C12	160.88 (11)
N6—C2—N2—C3	178.78 (12)	O1—Zn1—N5—C16	−102.31 (13)
C2—N2—C3—N3	0.9 (2)	O1^i^—Zn1—N5—C16	−19.44 (13)
C2—N2—C3—N8	179.95 (12)	O10—Zn1—N5—C12	−81.38 (13)
N1—C1—N3—C3	−8.0 (2)	O10—Zn1—N5—C16	98.30 (14)
N4—C1—N3—C3	166.17 (11)	N1—C2—N6—C17	−3.09 (19)
N2—C3—N3—C1	3.7 (2)	N1—C2—N6—C24	−165.36 (12)
N8—C3—N3—C1	−175.39 (12)	N2—C2—N6—C17	175.86 (12)
N1—C1—N4—Zn1	99.82 (11)	N2—C2—N6—C24	13.59 (18)
N1—C1—N4—C4	−153.32 (12)	C2—N6—C17—C18	121.40 (15)
N1—C1—N4—C11	−10.25 (17)	C2—N6—C17—C22	−56.99 (19)
N3—C1—N4—Zn1	−75.03 (11)	C24—N6—C17—C18	−76.76 (18)
N3—C1—N4—C4	31.83 (16)	C24—N6—C17—C22	104.84 (16)
N3—C1—N4—C11	174.90 (11)	N6—C17—C18—O2	−0.3 (2)
Zn1^i^—Zn1—N4—C1	121.41 (7)	N6—C17—C18—C19	−179.01 (13)
Zn1^i^—Zn1—N4—C4	1.58 (8)	C22—C17—C18—O2	178.12 (14)
Zn1^i^—Zn1—N4—C11	−118.40 (7)	C22—C17—C18—C19	−0.6 (2)
O1—Zn1—N4—C1	110.80 (8)	O2—C18—C19—C20	−176.18 (14)
O1^i^—Zn1—N4—C1	144.67 (9)	C17—C18—C19—C20	2.6 (2)
O1—Zn1—N4—C4	−9.03 (7)	C18—C19—C20—C21	−2.1 (2)
O1^i^—Zn1—N4—C4	24.84 (13)	C19—C20—C21—C22	−0.6 (2)
O1—Zn1—N4—C11	−129.01 (8)	C19—C20—C21—C23	−179.86 (16)
O1^i^—Zn1—N4—C11	−95.15 (11)	C20—C21—C22—C17	2.6 (2)
N5—Zn1—N4—C1	−134.86 (8)	C23—C21—C22—C17	−178.07 (15)
N5—Zn1—N4—C4	105.31 (8)	N6—C17—C22—C21	176.34 (13)
N5—Zn1—N4—C11	−14.68 (8)	C18—C17—C22—C21	−2.1 (2)
O10—Zn1—N4—C1	−1.61 (8)	C2—N6—C24—C25	−75.79 (16)
O10—Zn1—N4—C4	−121.44 (8)	C17—N6—C24—C25	121.81 (14)
O10—Zn1—N4—C11	118.57 (8)	N6—C24—C25—C26	128.15 (15)
Zn1—N4—C4—C5	5.41 (13)	N6—C24—C25—N7	−53.93 (17)
Zn1—N4—C4—C9	−172.18 (11)	C24—C25—C26—C27	176.35 (14)
C1—N4—C4—C5	−99.81 (15)	N7—C25—C26—C27	−1.4 (2)
C1—N4—C4—C9	82.59 (15)	C25—C26—C27—C28	0.3 (2)
C11—N4—C4—C5	116.69 (14)	C26—C27—C28—C29	1.0 (3)
C11—N4—C4—C9	−60.91 (16)	C27—C28—C29—N7	−1.2 (3)
N4—C4—C5—O1	3.88 (19)	C24—C25—N7—C29	−176.73 (14)
N4—C4—C5—C6	−177.27 (12)	C26—C25—N7—C29	1.2 (2)
C9—C4—C5—O1	−178.56 (12)	C28—C29—N7—C25	0.2 (3)
C9—C4—C5—C6	0.3 (2)	N2—C3—N8—C30	−16.9 (2)
C4—C5—O1—Zn1	−14.20 (17)	N2—C3—N8—C37	−179.36 (13)
C4—C5—O1—Zn1^i^	−156.31 (10)	N3—C3—N8—C30	162.23 (13)
C6—C5—O1—Zn1	167.00 (10)	N3—C3—N8—C37	−0.23 (19)
C6—C5—O1—Zn1^i^	24.9 (2)	C3—N8—C30—C31	129.60 (17)
Zn1^i^—Zn1—O1—C5	−153.10 (12)	C3—N8—C30—C35	−52.2 (2)
N4—Zn1—O1—Zn1^i^	165.58 (5)	C37—N8—C30—C31	−68.1 (2)
N4—Zn1—O1—C5	12.48 (9)	C37—N8—C30—C35	110.11 (17)
O1^i^—Zn1—O1—Zn1^i^	0.0	N8—C30—C31—O3	−3.0 (3)
O1^i^—Zn1—O1—C5	−153.10 (12)	N8—C30—C31—C32	177.53 (17)
N5—Zn1—O1—Zn1^i^	95.12 (5)	C35—C30—C31—O3	178.76 (19)
N5—Zn1—O1—C5	−57.98 (11)	C35—C30—C31—C32	−0.7 (3)
O10—Zn1—O1—Zn1^i^	−100.96 (5)	C30—C31—C32—C33	0.5 (3)
O10—Zn1—O1—C5	105.95 (10)	O3—C31—C32—C33	−178.9 (2)
C4—C5—C6—C7	2.0 (2)	C31—C32—C33—C34	0.1 (4)
O1—C5—C6—C7	−179.19 (14)	C32—C33—C34—C35	−0.6 (3)
C5—C6—C7—C8	−2.1 (2)	C32—C33—C34—C36	179.7 (2)
C6—C7—C8—C9	0.0 (2)	N8—C30—C35—C34	−178.04 (15)
C6—C7—C8—C10	179.56 (15)	C31—C30—C35—C34	0.2 (3)
N4—C4—C9—C8	175.07 (12)	C33—C34—C35—C30	0.5 (3)
C5—C4—C9—C8	−2.5 (2)	C36—C34—C35—C30	−179.80 (19)
C7—C8—C9—C4	2.3 (2)	C3—N8—C37—C38	−73.87 (17)
C10—C8—C9—C4	−177.28 (14)	C30—N8—C37—C38	123.35 (15)
Zn1—N4—C11—C12	20.18 (13)	N8—C37—C38—C39	125.93 (16)
C1—N4—C11—C12	126.93 (13)	N8—C37—C38—N9	−54.71 (17)
C4—N4—C11—C12	−89.40 (14)	C37—C38—C39—C40	178.78 (16)
N4—C11—C12—C13	165.30 (13)	N9—C38—C39—C40	−0.5 (3)
N4—C11—C12—N5	−18.10 (18)	C38—C39—C40—C41	−0.3 (3)
C11—C12—C13—C14	176.79 (15)	C39—C40—C41—C42	0.9 (3)
N5—C12—C13—C14	0.3 (2)	C40—C41—C42—N9	−0.7 (3)
C12—C13—C14—C15	−0.3 (3)	C37—C38—N9—C42	−178.61 (16)
C13—C14—C15—C16	0.3 (3)	C39—C38—N9—C42	0.7 (3)
C14—C15—C16—N5	−0.3 (3)	C41—C42—N9—C38	−0.1 (3)
C11—C12—N5—Zn1	2.92 (18)	O11—N10—O10—Zn1	−178.20 (14)
C11—C12—N5—C16	−176.76 (14)	O12—N10—O10—Zn1	1.63 (18)
C13—C12—N5—Zn1	179.46 (11)	Zn1^i^—Zn1—O10—N10	138.72 (9)
C13—C12—N5—C16	−0.2 (2)	N4—Zn1—O10—N10	−96.31 (10)
C15—C16—N5—Zn1	−179.46 (17)	O1—Zn1—O10—N10	−176.11 (10)
C15—C16—N5—C12	0.2 (3)	O1^i^—Zn1—O10—N10	98.28 (10)
Zn1^i^—Zn1—N5—C12	121.56 (11)	N5—Zn1—O10—N10	−17.26 (14)

Symmetry codes: (i) −*x*+1, −*y*, −*z*+1.

### Hydrogen-bond geometry (Å, °)

**Table:**

*D*—H···*A*	*D*—H	H···*A*	*D*···*A*	*D*—H···*A*
O2—H2···N7	0.84	1.93	2.745 (2)	164
O3—H3···N9	0.84	1.87	2.688 (2)	164
O1w—···.O3	.	.	2.676 (3)	.
O1w—···.O3^ii^	.	.	2.676 (3)	.

Symmetry codes: (ii) −*x*+1, *y*, −*z*+1/2.
